# Prevalence and Predictors of Postoperative Depression and Anxiety After Anterior Cruciate Ligament Reconstruction

**DOI:** 10.7759/cureus.45714

**Published:** 2023-09-21

**Authors:** Caitlin W Conley, Austin V Stone, Gregory S Hawk, Katherine L Thompson, Mary L Ireland, Darren L Johnson, Brian W Noehren, Cale A Jacobs

**Affiliations:** 1 Orthopedic Surgery and Sports Medicine, University of Kentucky, Lexington, USA; 2 Statistics, University of Kentucky, Lexington, USA; 3 Physical Therapy, University of Kentucky, Lexington, USA; 4 Orthopaedic Surgery, Brigham and Women’s Hospital, Boston, USA

**Keywords:** re-injury, acl tears, emotional wellbeing, mood disorder, knee

## Abstract

Purpose: Preoperative mood disorders influence postoperative outcomes after anterior cruciate ligament (ACL) reconstruction (ACLR), but the prevalence and risk factors associated with postoperative depression/anxiety development remain unknown. The purposes of this study were to quantify the prevalence of postoperative diagnoses of depression or anxiety following ACLR in patients under the age of 25 and assess the interplay between patient sex and ACL reoperation on the prevalence of a depression or anxiety diagnosis following ACLR.

Methods: ACLR patients under the age of 25 years old were identified in the Truven Healthcare Marketscan database. Patients with incomplete coverage +/- one year of the index surgical procedure were excluded. Patients were categorized by the presence of preoperative, postoperative, or no depression/anxiety using the International Classification of Diseases, Ninth Revision (ICD-9) codes. We compared patient demographics and reoperation rates following the index ACLR between the depression and anxiety categories. Additionally, logistic regression was fit to assess the interaction between sex and either ipsilateral or contralateral ACL surgery on postoperative depression/anxiety diagnosis.

Results: Of the 42,174 patients, 10.7% had a new depression/anxiety diagnosis after ACLR. Postoperative depression/anxiety was nearly twice as prevalent for females (F: 14.4%, M: 7.6%) despite having similar rates of secondary ACLR (F: 15.5%, M: 13.0%). Those with postoperative depression/anxiety had a considerably greater prevalence of reoperation (18.8%) than those without depression/anxiety (13.7%) and those with pre-existing preoperative depression/anxiety (12.9%). Sex and reoperation were independently associated with postoperative depression/anxiety diagnosis.

Conclusion: Female sex and secondary ACL surgery are independently associated with an increased prevalence of postoperative depression/anxiety. Nearly one in seven young females are diagnosed with depression/anxiety after ACLR. Similarly, a greater proportion of patients who suffer a secondary ACL surgery are subsequently diagnosed with depression/anxiety. The orthopedic community must be cognizant of the increased risk of postoperative depression/anxiety for females and those who suffer a secondary ACL surgery, and screening for depression/anxiety in these at-risk populations with referrals to mental health professionals may be warranted.

## Introduction

A greater emphasis is now being placed on evaluating psychological factors in patients undergoing anterior cruciate ligament (ACL) reconstruction (ACLR) to optimize the recovery process [1]. Progress has been made in the evaluation of psychological factors that influence postoperative outcomes, such as patient-reported outcomes, functional outcomes, and symptomatology from the knee joint [1]. However, despite up to 42% (n=27) of patients undergoing ACL surgery reporting major depressive disorder symptoms [2] and around a quarter reporting anxiety long term [3], we still do not understand what factors may contribute to the postoperative development of mood disorders [4].

Clinicians would benefit from having additional information on factors that may predispose a patient to develop a mood disorder postoperatively, as the presence of mood disorder symptoms can influence a patient’s recovery to varying degrees. Symptoms such as less motivation, fatigue, diminished interest in activities, psychomotor agitation, and inability to concentrate can negatively impact the already lengthy postoperative recovery and rehabilitation process [5,6]. Furthermore, depressive symptoms negatively influence postoperative outcomes used to assess postoperative recovery [2]. While less is known about anxiety, it is present in orthopedic patients post-injury [7] and can affect postoperative recovery such as return to sport. Given the potential rehabilitation implications outlined above, investigating red flag factors that can assist clinicians in early identification and thereby direct appropriate referrals is essential [7].

Sex and re-injury are two variables that specifically warrant further investigation. Females are at a higher risk for both ACL injuries [8] and mood disorders, such as depression [9]. When comparing psychological distress between sexes, females post-ACL surgery report that their emotions were influenced by improvements or setbacks in rehabilitation [10]. Female athletes are also twice as likely to report experiencing mild-to-moderate depressive symptoms when compared to males [9]. However, it is widely accepted that patients post-ACLR undergo a wide range of psychosocial responses during their recovery [11]. Thus, while females may be more likely to report depressive symptoms, it is unknown how often these symptoms transition into a clinical diagnosis, rather than being a transient mood state.

Injury is a risk factor for depressive symptoms in athletes [4,12]. The initial risk factor is further complicated by the risk that 5.8-16% of patients will experience a contralateral or ipsilateral ACL tear following the index ACLR [13]. Females are specifically at greater risk of sustaining a re-injury [8]. This risk of re-injury is prominent during the recovery process, as fear of re-injury has commonly been documented to influence the return to sport and the recovery process [14]. While many studies document negative outcomes associated with patients’ fear of re-injury [14], there is little information on the effect of a second injury on a patient's mental state. Reoperation negatively influences patient-reported outcomes [15] and recovery [16], which suggests that reoperation could have different implications in the presence of depression than the original injury.

The purposes of this study were to quantify the prevalence of postoperative diagnoses of depression or anxiety following ACLR in patients under the age of 25 and assess the interplay between patient sex and ACL reoperation on the prevalence of a depression or anxiety diagnosis following ACLR. We hypothesized that there would be a higher prevalence of a depression or anxiety diagnosis after ACLR and that the prevalence would be higher in females and those who experience a secondary ACLR (revision or contralateral ACLR).

## Materials and methods

The Truven Health Marketscan database was used to identify patients who underwent ACLR. The Truven database comprises over 135 million unique individuals from Marketscan Commercial claims and encounters and Medicare Supplemental databases (Truven Health Copyright© 2012, 2017 Truven Health Analytics Inc). The data from the Truven database do not include identifiable information and are provided through an honest broker; thus, this study is not considered human research requiring IRB approval. Age > 25 was an exclusion criterion due to the reduced risk of re-injury for ACLR patients in this age group [17]. Incomplete insurance coverage was selected as an exclusion criterion to eliminate confounding opioid prescriptions and associated healthcare costs.

To establish the prevalence of a depression or anxiety diagnosis, eligible patients were screened for insurance claims related to a depression or anxiety diagnosis. The specific International Classification of Diseases, Ninth Revision (ICD-9) codes, used to identify a depression or anxiety diagnosis, are presented in Table 1 [18,19]. Depression and anxiety diagnoses were categorized based on the time of diagnosis as preoperative, postoperative, or absent. Lastly, secondary ACL injury (contralateral or ipsilateral) and the need for subsequent ACLR were identified using CPT code 29888 occurring after the index ACLR. Since laterality is not included in ICD-9 codes, it was not possible to determine if the secondary ACL operation was a revision or a contralateral injury.

**Table 1:** ICD-9 Codes Used to Identify a Psychological Diagnosis

Code	Diagnosis
296	Episodic mood disorders
298	Other nonorganic psychoses
300	Anxiety, dissociative and somatoform disorders
309	Adjustment reaction
311	Depressive disorder, not elsewhere classified

Statistical analysis

Summary statistics were calculated from the Truven database to compare patient demographic variables across three groups: those with pre-existing depression or anxiety, those with a new postoperative diagnosis of depression or anxiety, and those without any diagnosis of depression or anxiety. For quantitative variables that were not normally distributed (time to reoperation and time to depression or anxiety diagnosis between sexes), data were summarized as median and interquartile range (IQR); otherwise, mean +/- standard deviation was reported. Due to the extremely large sample sizes in this study population, traditional hypothesis testing and p-values are not appropriate to report, since even small differences between groups will appear to be highly significant despite not being clinically meaningful [18]. For example in our dataset, age differences would have been found to be statistically significant between the three groups (p<0.01). However, the mean age of the three groups were separated by only one year (no depression or anxiety diagnosis: 16.4 + 3.8yrs, pre-op depression or anxiety diagnosis: 16.9 + 4.1yrs, and post-op depression or anxiety diagnosis: 15.7 + 3.7yrs) and likely not clinically meaningful.

A logistic regression model for postoperative depression or anxiety diagnosis was fit to assess the appropriateness of a two-way interaction between sex and ACL reoperation (defined as surgery to either the ipsilateral or contralateral knee). All analyses were completed using R, version 3.6.1 (R Foundation for Statistical Computing; Vienna, Austria).

## Results

A total of 82,962 patients underwent ACLR between January 2009 and September 2014 identified by CPT code 29888. Of ACLRs, 42,174 patients (50.8%) were <25 years old and had complete insurance coverage in the year prior to and following the index ACLR procedure to include in the study. There were more males (n=23,208, 55%) than females (n=18,966, 45%) in our sample, with an average age of 16 years old. A total of 8,901 patients (21.1%) had a diagnosis of depression or anxiety: 10.4% (n=4390) with a preoperative depression or anxiety diagnosis and 10.7% (n=4511) with a new postoperative depression or anxiety diagnosis after the index ACLR (Table 2). A larger proportion of females had either a preoperative (52.3%, n=2,294) or postoperative (60.7%, n=2,740) diagnosis of depression or anxiety compared to males (47.7%, n=2,096 preoperative; and 39.3%, n=1,771 postoperative).

**Table 2:** Demographic Characteristics of Patients Undergoing ACL Reconstruction by Depression or Anxiety Diagnosis Data presented as mean +/- standard deviation or as counts and percentages where appropriate.

Variable		No Depression or Anxiety Diagnosis	Pre-op Depression or Anxiety Diagnosis	Post-op Depression or Anxiety Diagnosis
Number	N (%)	33,273 (78.9%)	4,390 (10.4%)	4,511 (10.7%)
Age	Years	16.4 + 3.8	16.9 + 4.1	15.7 + 3.7
Sex	Female	13,932 (41.9%)	2,294 (52.3%)	2,740 (60.7%)
	Male	19,341 (58.1%)	2,096 (47.7%)	1,771 (39.3%)
Re-operation	No	28,729 (86.3%)	3,825 (87.1%)	3,661 (81.2%)
	Yes	4,544 (13.7%)	565 (12.9%)	850 (18.8%)

Secondary ACL injury occurred in 14.13% (n=5,959) of the patients, on average, just over a year after the initial ACLR (Table 3). Patients with a postoperative depression or anxiety diagnosis had the largest percentage of secondary ACL injuries (18.8%, n=850), followed by 13.7% (n=4,544) of those patients without a depression or anxiety diagnosis and 12.9% (n=565) of those patients with a preoperative depression or anxiety diagnosis (Table 2). Of those patients with a secondary injury and postoperative depression or anxiety diagnosis, 77.8% (n=661) of the patients had their secondary ACLR (ipsilateral or contralateral) before the initial depression or anxiety diagnosis. 

The prevalence of a postoperative depression or anxiety diagnosis was considerably greater for females, with nearly double the prevalence as compared to their male counterparts (14.4%, n=2740 vs. 7.6%, n=1771) despite having similar rates of secondary ACLR (females=15.5%, n=2942 vs. males=13.0%, n=3017; Table 3). Even with the large sample size, a logistic regression model did not identify a significant interaction effect between sex and secondary ACLR, suggesting that sex and secondary ACLR are independently associated with postoperative depression or anxiety diagnosis.

**Table 3:** ACL Reoperation and Anxiety or Depression Diagnosis Between Sexes for Patients Undergoing ACL Reconstruction Data presented as medians and IQR or as counts and percentages where appropriate. ACL: Anterior Cruciate Ligament

Variable	Males	Females
Total Patients (%)	23,208 (55.0%)	18,966 (45.0%)
Patients Undergoing a Reoperation (%)	3,017 (13.0%)	2,942 (15.5%)
Time to ACL Reoperation (days)	414 (IQR = 243-714)	428 (IQR = 266-712)
Patients with a Pre-existing Depression or Anxiety Diagnosis (%)	2,096 (9.0%)	2,294 (12.1%)
Patients with a Postoperative Depression or Anxiety Diagnosis (%)	1,771 (7.6%)	2,740 (14.4%)
Time to Postoperative Depression or Anxiety Diagnosis (days)	557 (IQR = 236-1010)	765 (IQR = 384-1238)

## Discussion

The primary findings of this study were that the female sex and subsequent ACLR (ipsilateral or contralateral) are independently associated with increased postoperative diagnoses of depression or anxiety after the primary ACLR. Furthermore, in patients with postoperative depression or anxiety, over two-thirds had a diagnosis of depression or anxiety after the second ACL operation. This finding suggests that secondary ACLR may increase the likelihood of a depression or anxiety diagnosis. 

The unadjusted prevalence of a postoperative depression or anxiety diagnosis after ACLR was nearly twice as large for females under the age of 25 when compared to males. This finding is in line with both a meta-analysis that found female high-performance athletes are twice as likely to report depression symptoms than male high-performance athletes [9] and a systematic review that found female sex was a risk factor for depression in high-performance athletes [4]. This trend is not just common in athletes and those with ACL injuries but also true for other orthopedic procedures. Females had three times the odds of having diagnosed postoperative depression after total knee arthroplasty compared to males [20].

Our study added to the literature by examining the interplay of sex and re-injury on postoperative depression. Our hypothesis was not supported as we did not find a relationship between sex and re-injury. Given the independent relationship between postoperative depression and female sex, there is a clear need to monitor female ACLR patients for a change in depression or anxiety symptoms. Clinicians can quickly evaluate patients’ symptoms by using a short four-item questionnaire, such as the Patient Health Questionnaire (PHQ-4) [21]. It may be beneficial to conduct this monitoring over the course of the rehabilitation process as daily stress and pain have been positively associated with ACL patient’s negative mood state after surgery [22]. In elite athletes specifically, high levels of depressive symptoms have been related to high levels of chronic stress, negative coping strategies, and negative stress-recovery states [12]. Furthermore, while males and females have demonstrated similar levels of functional performance [23], perceived function [23,24], fear of movement (Tampa Scale of Kinesiophobia) [24], and readiness for return to sport (ACL Return to Sport After Injury Scale) before returning to athletic competition [24], the rate of returning to sport is lower for females [23]. The combination of these previous findings and our current results highlights a need to develop sex-specific interventions, which include mental health components to address the disparity in knee-related outcomes for female ACLR patients.

There also appears to be a psychological impact of reinjury as secondary ACLR (ipsilateral or contralateral) was identified as being independently associated with a postoperative depression or anxiety diagnosis. Furthermore, there was a greater prevalence of secondary ACLR among those with a postoperative depression or anxiety diagnosis, and in most cases, the secondary ACLR preceded the mood disorder diagnosis. This finding builds on a recent systematic review, which found that elite athletes had a higher risk of depression if they experienced injuries [4]. However, the review did not look at the types of injuries or if the injury was a re-injury. When considering the rehabilitation process for the second ACL injury, our results in conjunction with this finding highlight a critical component of recovery given that psychological factors have been shown to be related to poor outcomes, such as range of motion early in rehabilitation [11] and return to sport in ACL patients [25]. These findings equip clinicians with additional information and factors that could negatively influence the rehabilitation process.

These findings also underscore the importance of secondary injury prevention. Much like the female sex, the need for additional surgery has been associated with worsening symptoms between two and six years after ACLR, inferior 10-year patient-reported outcomes, and a greater proportion of patients that failed to achieve a patient-acceptable symptom state requiring reoperation [15,26]. When patients who underwent an ACLR are grouped by their postoperative recovery trajectories (those who improved at one year postoperative and maintained the improvements up to two years postoperative, those who continued to improve up to two years postoperative, and those who improved one year postoperative, but then got worse at two years postoperative), statistically more patients had undergone a revision in the group who improved but got worse at two years [16]. A lengthy postoperative rehabilitation process and/or the risk of a secondary ACL injury have been suggested as factors that increase the risk of developing mood disorders such as depression or anxiety following ACLR [2,27]. Considering that 23% of athletes (n=170) under the age of 25 who return to sport sustain a subsequent secondary ACL injury [28], it is imperative to develop and implement secondary injury prevention programs, such as the FIFA-11 for soccer [29], to lessen the risk of inferior mental health and knee-related outcomes.

Ultimately, regardless of sex or secondary ACLR, nearly 10% of patients under the age of 25 were diagnosed with depression or anxiety after ACLR. To date, other studies have employed questionnaires to quantify psychiatric symptoms. For example, Garcia et al. utilized a validated patient-reported outcome instrument to quantify depressive symptoms and reported that 27/64 patients (42%) demonstrated significant preoperative depressive symptoms following ACL injury [2]. While 42% (n=27) of the overall participants reported preoperative depressive symptoms, only 5/27 (18.5%) of those with depressive symptoms had been diagnosed with a mental health disorder [2]. Interestingly, the prevalence of diagnosis reported by Garcia et al. (18.5%, n=5)) is closer to the preoperative prevalence we reported (10.4%, n=4,390)), as well as the incidence of overall prevalence of major depressive episodes for adolescents (14.7%) or young adults between the ages of 18 and 25 (18.6%) [30]. This discrepancy between diagnosis and scores from self-reported depression questionnaires highlights the speculated gap in the presence of mental health symptoms and mental health diagnosis or treatment. Mental health problems are underreported, undertreated, and understudied in athletes [4,25]. Thus, it is suspected that, while the use of an insurance claims database provided a beneficial summary of the patients seeking treatment, the 10% incidence in the current study is likely an underestimate of depression or anxiety symptoms that patients experience after ACLR.

Limitations

There are limitations to our study. Diagnosis codes were utilized to identify the presence of depression or anxiety. Classifying patients in this manner does not reflect the patient’s symptom state at the time of diagnosis. Some patients may be currently under successful care, while other patients may be more negatively affected by their depression or anxiety. Furthermore, it is possible that patients classified in the no depression or anxiety group had underlying symptoms of depression or anxiety that were undiagnosed during either the pre- or postoperative period. We were also unable to assess a patient’s postoperative activity level or whether they returned to sports. The lack of sex difference in reinjury rates in larger studies such as ours that do not specifically target athletes may potentially mask sex-related reinjury rates for female athletes. Another limitation is that we were unable to differentiate whether a secondary ACLR was performed due to ipsilateral graft failure or contralateral injury as only ICD-9 codes were available. Lastly, while the Truven database yields a sizeable sample size, this sample may not be reflective of individuals in other insurance plans, government assistance programs, or those who are uninsured.

## Conclusions

Female sex and secondary ACLR are independently associated with an increased prevalence of postoperative depression and anxiety. Nearly one in seven female patients are diagnosed with depression or anxiety after ACLR, which is likely an underestimate as mental health problems are underreported, undertreated, and understudied. Similarly, a greater proportion of patients who suffer a secondary ACLR are subsequently diagnosed with depression or anxiety. The orthopedic community must be cognizant of the increased risk of postoperative mood disorders for females and those who suffer a secondary ACLR, and these results suggest that screening for depression and anxiety for these at-risk populations with referral to mental health professionals for appropriate care may be warranted.
